# Gender Differences in the Correlations between Childhood Trauma, Schizotypy and Negative Emotions in Non-Clinical Individuals

**DOI:** 10.3390/brainsci12020186

**Published:** 2022-01-29

**Authors:** Elizabeth H. X. Thomas, Susan L. Rossell, Caroline Gurvich

**Affiliations:** 1 Department of Psychiatry, The Alfred Hospital and Central Clinical School, Monash University, Melbourne, VIC 3004, Australia; caroline.gurvich@monash.edu; 2 Centre for Mental Health, School of Health Sciences, Swinburne University, Hawthorn, VIC 3122, Australia; srossell@srossell.com; 3 St. Vincent’s Mental Health, St. Vincent’s Hospital, Fitzroy, VIC 3065, Australia

**Keywords:** schizophrenia spectrum, early life adversity, depression, anxiety, stress

## Abstract

Early life trauma has a negative impact on the developing brain, and this can lead to a wide range of mental illnesses later in life. Childhood trauma is associated with increased psychotic symptoms and negative emotions such as depressive, anxiety, and stress symptoms in adulthood. Childhood trauma has also been shown to influence sub-clinical ‘schizotypy’ characteristics of psychosis in the general population. As it has been reported that mental health outcomes after early life trauma exposure are influenced by gender, the current study aimed to investigate the gender differences in the relationship between childhood trauma, schizotypy and negative emotions. Sixty-one non-clinical participants (33 men and 28 women) aged between 18 and 45 completed self-report questionnaires to measure early life trauma, schizotypy and negative emotions. Despite similar levels of childhood trauma in men and women, early life trauma in women was associated with increased schizotypy personality characteristics (Cognitive Disorganisation) and increased depression, anxiety and stress later in life, but no correlations were observed in men. Our findings suggest that the sociocultural and biological processes affected by early life adversities may differ between the genders. Women may be more vulnerable to the influence of childhood trauma, which may be associated with increased psychopathology later in life.

## 1. Introduction

Research shows that schizophrenia symptoms and the course of illness differ between men and women; for instance, men have a higher prevalence of negative symptoms, lower functioning and an earlier symptom onset of three-to-five years compared to women [1]. In contrast, women often present with more depressive and affective symptoms [1]. These differences in symptom onset, course and presentation suggest men and women may experience different responses to schizophrenia risk factors, which involve different sociocultural (gender) and biological processes [2].

One gender-related factor underlying the development of schizophrenia symptomatology could be early life trauma. A higher prevalence of childhood trauma has been observed in first-episode psychosis patients compared to healthy controls, and it has been suggested that psychosis may result from trauma experienced in childhood [3]. Childhood trauma can lead to the dysregulation of the HPA axis and, according to the neural diathesis-stress model of psychosis, may alter the cortisol feedback loop and subsequently increase vulnerability to developing psychotic and depressive symptoms [4,5]. Research suggests that stress contributes to HPA dysfunction in women more than men [6], which may explain gender differences in outcomes following exposure to childhood trauma. In individuals with psychosis, childhood trauma and/or adversity has been associated with poorer physical health (such as cardiovascular disease and epilepsy) [7] and negative symptoms [8] in men, and increased depression in women [7,8,9].

While gender differences in schizophrenia symptomatology have been previously observed [1], research has been inconsistent [10]. This may reflect other factors that differ between men and women with schizophrenia, such as higher alcohol and cannabis abuse in men [11,12], which are often not controlled for in research [2]. This can be addressed by using schizotypy (sub-clinical characteristics of psychosis present in the general population that reflect a milder form of schizophrenia symptoms [13]); this allows for the exploration of gender differences in the absence of such confounders. Using schizotypy as a model for schizophrenia also provides a useful approach to investigate the trauma–schizotypy relationship; the recall of traumatic events in a non-clinical population assessed for schizotypy is less likely to be affected by psychotic symptoms, cognitive problems or the medication effects associated with schizophrenia.

A review of 25 studies investigating childhood trauma and schizotypy observed that all studies found an association between schizotypy and at least one type of trauma, mainly physical abuse, sexual abuse or neglect [14]. Two studies have shown that associations between childhood trauma and schizotypy differ between genders. Berenbaum et al. [15] observed schizotypy traits in individuals with schizotypal personality disorder to be more strongly associated with childhood trauma in men, and more associated with post-traumatic stress disorder (PTSD) criteria in women, while Toutountzidis et al. [16] observed relationships between early life trauma and schizotypal traits in women but not in men. Two studies did not observe any gender differences in the trauma–schizotypy relationship [17,18]. It should be noted that these aforementioned studies have either investigated schizotypy in the context of schizotypal personality disorder [15,17,18], or used schizotypy inventories designed to capture traits relevant to schizotypal personality disorder specifically [16]. As such, the effect of gender on the relationship between trauma and schizotypy traits has not been investigated in the general population. It is important to distinguish schizotypy research from studies in schizotypal personality disorder; while patients with a diagnosis of schizotypal personality disorder are likely to have high levels of schizotypy, not all individuals with high schizotypy will meet diagnostic criteria for schizotypal personality disorder.

We aimed to explore gender differences in the relationship between schizotypy and trauma outside the context of schizotypal personality disorder using a cross-sectional correlational approach. As depression and anxiety are expressed at higher rates in females than males in the general population, and because women with schizophrenia present more with mood symptoms rather than positive and negative symptoms, we also investigated the association between childhood trauma and depression, anxiety and stress in both genders, which has not been explored alongside schizotypy.

## 2. Materials and Methods

### 2.1. Participants

Sixty-one non-clinical participants aged between 18 and 45 responded to advertisements and met the inclusion criteria of being fluent English language speakers. Participants were excluded if they met diagnostic criteria for a psychotic or affective disorder (as per the Mini-International Neuropsychiatric Interview (MINI) screening module criteria), a history of stroke, severe head injury, loss of consciousness or epilepsy or any significant visual impairment, other than refractive.

### 2.2. Self-Report Measures

All participants completed the Oxford-Liverpool Inventory of Feelings and Experiences (O-LIFE), a 104-item self-report questionnaire developed to measure schizotypy in non-clinical individuals [19]. The O-LIFE measures the three main dimensions of schizotypy: (1) the Unusual Experiences scale reflects positive schizophrenia symptomatology, (2) the Introvertive Anhedonia scale reflects negative symptomatology and (3) the Cognitive Disorganisation scale reflects disorganised symptoms of psychosis. The fourth factor, Impulsive Nonconformity, was excluded as it reflects traits found in personality disorders rather than features of schizophrenia and does not differ between patients and controls [13].

The Depression Anxiety Stress Scale (DASS) was used to measure the negative emotional states of depression, anxiety and tension/stress over the past week [20]. The short form consists of 21 items, and each of the subscales consists of seven items, with higher scores indicating higher emotional distress.

The Childhood Trauma Questionnaire-Short Form (CTQ-SF) was included to assess early life stress/adversity [21]. The short form version consists of 28 items capturing emotional, physical and sexual abuse as well as emotional and physical neglect that occurred in childhood.

As the current study investigates gender differences, it is important to note that strong measurement invariance across gender has been observed for the O-LIFE [22], DASS-21 [23] and CTQ-SF [24].

### 2.3. Statistical Analysis

Data analyses were performed using IBM SPSS version 26. As variables violated the assumptions of normality, Mann–Whitney U-tests were conducted to compare gender differences. Spearman’s rho correlations between childhood trauma, schizotypy and negative emotion were conducted separately for men and women. Bonferroni corrections were made to account for multiple comparisons; *p* ≤ 0.005 for the Mann–Whitney U-tests to account for 11 comparisons and *p* ≤ 0.002 for the Spearman’s rho correlations to account for 30 correlations in men and women. A correlation heatmap was generated using GraphPad Prism version 9.1.2.

## 3. Results

Mean and median age, trauma, schizotypy and negative emotion measures are described in Table 1 according to gender. There were no significant differences in gender for variables except for Cognitive Disorganisation, which was significantly higher in women.

**Figure 1:**
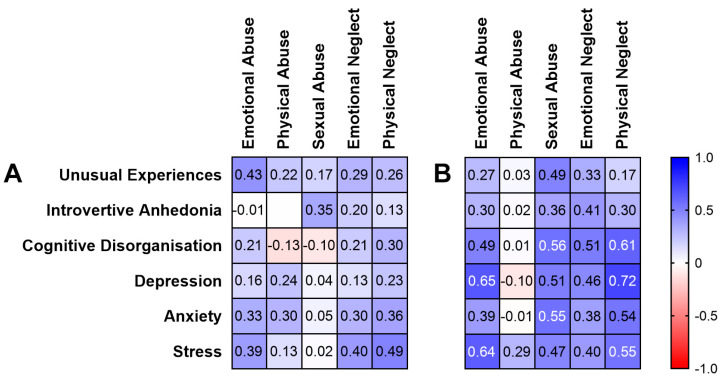
Spearman’s rho correlation heatmap of childhood trauma, schizotypy and negative emotion measures in (**A**) men and (**B**) women. Cell shading represents relationship direction and strength (blue = positive, red = negative). White text indicates statistical significance (≤0.002 after Bonferroni correction).

Looking at the correlation heatmap (Figure 1), the correlation coefficients in women were higher than in men overall (indicated by cell shading representing the relationship direction and strength). When examining the correlations, seven were significant after Bonferroni correction in women (Figure 1), but no relationships between childhood trauma, schizotypy and negative emotions were observed in men. 

In women, Cognitive Disorganisation was significantly positively correlated with sexual abuse (rho = 0.56, *p* = 0.002; 95% CI, 0.21–0.78) and physical neglect (rho = 0.61, *p* < 0.001; 95% CI, 0.28–0.82). With regards to negative emotion in women, emotional abuse positively correlated with depression (rho = 0.65, *p* < 0.001; 95% CI, 0.33–0.84) and stress (rho = 0.64, *p* < 0.001; 95% CI, 0.31–0.83). Physical neglect was also significantly positively correlated with depression (rho = 0.72, *p* < 0.001; 95% CI, 0.44–0.87) and stress (rho = 0.55, *p* = 0.002; 95% CI, 0.20–0.78). Sexual abuse also positively correlated with anxiety (rho = 0.55, *p* = 0.002; 95% CI, 0.20–0.78).

## 4. Discussion

### 4.1. Childhood Trauma and Disorganised Schizotypy

Our study found that the Cognitive Disorganisation schizotypy factor, which captures poor attention, concentration, decision-making and social cognition, was positively correlated with physical neglect and sexual abuse in women, highlighting that schizotypy is not associated with a single type of trauma. This is consistent with the increased self-reports of memory disorganisation [25] and poorer cognitive functioning [26] in individuals who have experienced childhood trauma.

Of the two observed significant relationships in our study, Cognitive Disorganisation was most strongly correlated with physical neglect, consistent with schizophrenia research showing the most robust relationship between physical neglect in childhood and functional and social impairment [27]. The Cognitive Disorganisation factor has not been previously explored in these studies looking at gender differences in the trauma–schizotypy relationship, and as such, our findings require replication.

While childhood trauma was associated with schizotypy in women, this was not observed in men. This is consistent with Toutountzidis et al. [16], but not with other studies [15,17,18]. As our study is the first to look at the trauma–schizotypy relationship outside of the schizotypal personality disorder context, this warrants further investigation.

### 4.2. Childhood Trauma and Negative Emotions

Unexpectedly, depression, anxiety and stress levels did not significantly differ between men and women. However, trauma positively correlated with depression, anxiety, and stress in women but not in men, consistent with previous observations of a relationship between higher CTQ scores and depressive symptoms in women during early psychosis [8,9]. In women, emotional abuse and physical neglect were associated with both depression and stress. As with schizotypy, negative emotion does not appear to be associated with one specific type of trauma. Sexual abuse was associated with anxiety only; a possible explanation is that women who have experienced sexual abuse may have more self-blame compared to other types of trauma, and women who report more self-blame have elevated levels of trait anxiety not observed in men [28].

The trauma–mood relationship observed in women but not in men could also potentially be explained by the different coping mechanisms employed by men and women. Cognitive emotion regulation strategies have been shown to mediate the relationship between childhood trauma and depression and anxiety in adulthood across both genders [29]. Emotional coping is associated with higher levels of depression, anxiety and distress compared to problem-focused coping [30], with women tending to use the former and men tending to use the latter [31]. Women also tend to internalise their pain, which later manifests itself as depression and anxiety [32]. Men tend to externalise their pain, which manifests more as behavioural problems and alcohol and substance abuse [32]. This also explains previous findings of increased alcohol intake in men who have experienced childhood trauma [9].

### 4.3. Gender Differences in the HPA Axis as an Underlying Mechanism

Increased HPA axis activity has been reported in both schizophrenia [5] and depression [33]. In addition, HPA axis dysfunction in clinical high-risk populations has been shown to resemble those in psychosis [5]. In line with this research, our findings of a relationship between trauma and Cognitive Disorganisation can be explained by altered HPA axis function, as there have been previous associations between dysfunctional HPA axis activity, altered cortisol levels and both increased disorganisation symptom severity in schizophrenia [34] and poorer cognitive performance in both schizophrenia and depression [33,35,36]. Similarly, our significant findings with negative mood are also supported by previous associations between cortisol levels and depressive and anxiety symptoms in women [37].

Biologically, our significant findings in women but not in men may be explained by sex differences in the HPA axis. The HPA axis has been shown to be more reactive to stress in females compared to males [6]. This may be related to differences in sex hormone levels, regulated by the hypothalamic–pituitary–gonadal axis (such as circulating estrogen and progesterone) and their interaction with HPA axis function [6].

Sex differences in HPA axis function is demonstrated in both animal and clinical studies. Findings from animal studies have been consistent, showing higher glucocorticoid levels observed in females after stimulation of the HPA axis [10]. The interaction between sex with age and the developmental window influences outcomes after stress exposure [38], such as male-specific resilience to depressive-like pathology but a female-specific vulnerability to depression after early life stress [6,39]. However, the development of psychotic and depressive symptoms after trauma is determined by a combination of biological, psychological and social factors [40], not all of which can be explored in animal studies. While these can be better investigated in clinical research, findings are less consistent regarding biological mechanisms [10]. There has been research demonstrating that women with childhood trauma experiences [41], women with schizophrenia [42] and women with depression [37] have significantly different cortisol levels to men. However, whether women show a hyper- or hypoactive cortisol response (i.e., higher or lower levels) than men is uncertain, and likely depends on the stressor [37].

### 4.4. Limitations, Future Research and Clinical Implications

The findings of this study should be interpreted within the context of several limitations. Firstly, as individuals are likely to experience more than one type of trauma, the trauma types are likely intercorrelated, making it difficult to draw conclusions about the impact of specific types of trauma on schizotypy and negative emotion. Future research should investigate the effect of cumulative trauma, of both single and multiple trauma types across childhood and into adulthood. Our reported trauma scores were also relatively low, and our research needs to be replicated in a larger, broader, non-clinical sample with more individuals who have experienced childhood trauma.

Our study demonstrates the relationship between childhood trauma, disorganised schizotypy and negative emotion in women. This highlights the importance for individuals identified as high risk for psychosis with childhood trauma experiences to receive early interventions, such as trauma-focused cognitive–behavioural therapy, to address cognitive distortions related to the trauma. Our findings also indicate increased vulnerability in women to adverse childhood events and suggest that the sociocultural and biological mechanisms affected by early life adversities may differ between genders. This contributes to a growing body of work highlighting the importance of early life trauma in both schizotypy and mood disorders, and supports the development of gender-specific interventions to strengthen resilience.

## Figures and Tables

**Table 1:** Age, childhood trauma, schizotypy and negative emotion measures by gender.

	Men		Women		Test Statistic	
	Mean (SD)	Median (IQR)	Mean (SD)	Median (IQR)	U	*p*
Age	24.76 (4.35)	23.00 (6.00)	23.18 (4.04)	22.50 (4.00)	375.50	0.21
CTQ measures						
Emotional Abuse	6.24 (1.82)	5.00 (2.00)	8.11 (3.63)	7.00 (4.00)	315.00	0.03
Physical Abuse	5.55 (1.03)	5.00 (1.00)	6.18 (2.29)	5.00 (1.75)	425.00	0.51
Sexual Abuse	5.45 (1.50)	5.00 (0.00)	5.96 (2.01)	5.00 (1.00)	398.50	0.20
Emotional Neglect	8.27 (3.30)	7.00 (4.00)	8.81 (3.86)	8.00 (6.00)	417.00	0.67
Physical Neglect	6.41 (2.11)	5.00 (2.00)	6.32 (1.63)	5.00 (2.75)	437.00	0.86
Schizotypy measures						
Unusual Experiences	6.55 (6.29)	5.00 (8.50)	8.71 (6.43)	7.50 (11.50)	363.50	0.15
Introvertive Anhedonia	6.67 (5.01)	6.00 (6.00)	5.96 (4.09)	5.50 (6.75)	439.50	0.74
Cognitive Disorganisation	7.69 (5.47)	7.00 (7.50)	12.04 (5.76)	14.00 (10.00)	258.00	0.005 *
Negative emotion measures						
Depression	2.85 (4.40)	1.00 (3.00)	2.39 (2.38)	2.00 (2.75)	431.00	0.65
Anxiety	2.91 (3.19)	2.00 (3.00)	3.36 (3.46)	2.50 (4.75)	429.50	0.63
Stress	3.88 (3.69)	3.00 (3.50)	4.43 (3.39)	4.00 (5.75)	404.50	0.40

* *p* ≤ 0.005, significant after Bonferroni correction.

## Data Availability

Data can be requested from the authors.
